# The Role of Peer Specialists in Engaging Aging Adults in Recovery and Wellness Behaviors

**DOI:** 10.1093/geroni/igaa057.2153

**Published:** 2020-12-16

**Authors:** Amanda Peeples, Laura Wray

## Abstract

Peer specialists are individuals who have a lived experience with a mental health condition and who have received formal training to provide peer support and services to others. This symposium will examine the role of peer specialists in supporting aging adults’ recovery, quality of life, health behaviors, and health outcomes. Peer specialists have increasingly been integrated as formal service providers in health care systems in a wide variety of settings. In the Veterans Health Administration (VHA), over 1,110 Veterans work as peer specialists across the country. Findings from both VHA and community studies will be presented. First, Amanda Peeples will present findings from a mixed-methods evaluation study on the implementation of peer specialists to primary care teams in the VHA. Next, Anjana Muralidharan will present on the role of peer specialists in promoting health and wellness among aging veterans with serious mental illness using data from three VHA studies on peer-led interventions. Finally, Karen Fortuna will present on PeerTECH, a digital peer support self-management intervention that teaches older adults with mental health conditions how to co-manage psychiatric illness and chronic health conditions. Together, these three presentations will explore the ways in which “peerness” is defined in different populations and in diverse contexts. Laura Wray, geropsychologist and Executive Director of the VHA Center for Integrated Healthcare, will serve as discussant. She will comment on the role of peer specialists in supporting the recovery and wellness of aging persons in VHA and beyond.

